# Metabolic Reprogramming of *Klebsiella pneumoniae* Exposed to Serum and Its Potential Implications in Host Immune System Evasion and Resistance

**DOI:** 10.1021/acs.jproteome.4c00286

**Published:** 2024-10-03

**Authors:** Amanda Naiara Silva Moraes, Juliana Miranda Tatara, Rafael Lopes da Rosa, Franciele Maboni Siqueira, Guilherme Domingues, Markus Berger, Jorge Almeida Guimarães, Afonso Luís Barth, Patricia Orlandi Barth, John R. Yates, Walter Orlando Beys-da-Silva, Lucélia Santi

**Affiliations:** † Post-Graduation Program in Cellular and Molecular Biology, Federal University of Rio Grande do Sul., Porto Alegre, Rio Grande do Sul 91501-970, Brazil; ‡ Faculty of Veterinary, Federal University of Rio Grande do Sul, Porto Alegre, Rio Grande do Sul 91540-000, Brazil; § Bruno Born Hospital, Lajeado, Rio Grande do Sul 95900-010, Brazil; ∥ Center of Experimental Research, Clinical Hospital of Porto Alegre, Porto Alegre, Rio Grande do Sul 90035-903, Brazil; ⊥ Tick-Pathogen Transmission Unit, Laboratory of Bacteriology, National Institute of Allergy and Infectious Diseases, Hamilton, Montana 20892, United States; # Bacterial Resistance Research Laboratory, Clinical Hospital of Porto Alegre, Porto Alegre, Rio Grande do Sul 90035-903, Brazil; ∇ Department of Molecular Medicine, Scripps Research, La Jolla, California 92037, United States; ○ Faculty of Pharmacy, Federal University of Rio Grande do Sul, Porto Alegre, Rio Grande do Sul 90610-000, Brazil

**Keywords:** serum resistance, glyoxylate cycle, pyruvate, virulence, metabolic reprogramming

## Abstract

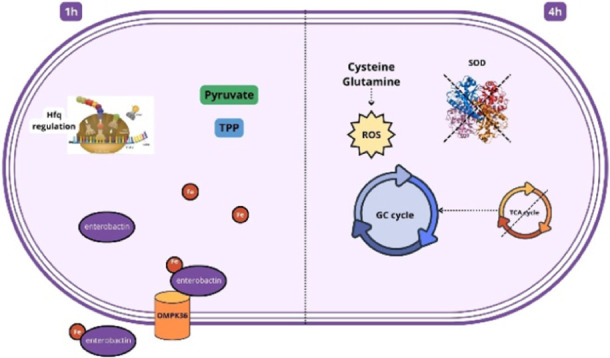

The aim of this study was to identify, using proteomics, the molecular alterations caused by human serum exposure to *Klebsiella pneumoniae* ACH2. The analysis was performed under two different conditions, native serum from healthy donors and heat-inactivated serum (to inactivate the complement system), and at two different times, after 1 and 4 h of serum exposure. More than 1,000 bacterial proteins were identified at each time point. Enterobactin, a siderophore involved in iron uptake, and proteins involved in translation were upregulated at 1 h, while the chaperone ProQ and the glyoxylate cycle were identified after 4 h. Enzymes involved in the stress response were downregulated, and the SOD activity was validated using an enzymatic assay. In addition, an intricate metabolic adaptation was observed, with pyruvate and thiamine possibly involved in survival and virulence in the first hour of serum exposure. The addition of exogenous thiamine contributes to bacterial growth in human serum, corroborating this result. During 4 h of serum exposure, the glyoxylate cycle (GC) probably plays a central role, and the addition of exogenous succinate suppresses the GC, inducing a decrease in serum resistance. Therefore, serum exposure causes important changes in iron acquisition, the expression of virulence factors, and metabolic reprogramming, which could contribute to bacterial serum resistance.

## Introduction

*Klebsiella pneumoniae* is a Gram-negative encapsulated bacterium present in the human microbiota from the gastrointestinal tract and nasopharynx.^1^ This pathogen belongs to the “ESKAPE” group, which comprises six highly virulent and antibiotic-resistant bacterial pathogens.^2^*K. pneumoniae* is known to be responsible for causing several diseases in humans, including urinary tract infections, pneumonia, and sepsis, and has become the most common pathogen responsible for nosocomial infections due to the identification of hypervirulent isolates and multi-resistance to most antimicrobials.^3^ In fact, an increased mortality rate has been observed for severe sepsis worldwide, with almost 20% of deaths caused by antibiotic-resistant *K. pneumoniae*.^4,5^ According to the World Health Organization (WHO), the development of new treatments and options to eradicate or minimize the mortality triggered by *K. pneumoniae* is considered an urgent need.^3^

Several strategies have been acquired by microorganisms to survive, counterattack, and evade the human immune system. While most microorganisms are susceptible to the microbicidal property of human serum, serum-resistance mechanisms, including metabolic shifting, virulence factors, and iron acquisition, have evolved. This includes capsule and superoxide dismutases, among others, which helps bacteria to exploit host resources and modulate and evade the immune surveillance.^6−9^ Siderophores can also act as toxins, modulating the immune system and activating mitophagy pathways in platelets.^10^ Although efforts are being made to understand the mechanisms involved in serum resistance, it is still poorly understood at the molecular level.

Omics approaches have been used as effective tools in the study of biological processes, which may be involved in the host’s response to infection, replication of pathogens in the host and disease progression, making it an extremely useful tool for understanding host–pathogen interactions.^11^ Concerning proteomics approaches, bottom-up proteomics can be used to provide impartial and sensitive measurements in order to characterize proteomes under pathogenic conditions. This analysis can be performed from the perspective of the pathogen or host, or even both, allowing us to understand the molecular complexities of the response of both, by leading to a deeper knowledge of the mechanisms of microbial virulence and significant new targets for future drug discovery.^12^

During infection, bacterial pathogens successfully sense, respond, and adapt to a myriad of harsh environments presented by the mammalian host. This remarkable level of adaptation requires a robust modulation of their physiological and metabolic features. Thus, an understanding of bacterial metabolism during pathogenesis and the metabolic pathways that are possibly activated is extremely important to circumvent it for humans’ benefit.

Here, using proteomics, we evaluated molecular alterations in *K. pneumoniae* caused by serum exposure. By comparing the effects of native serum and heat-inactivated serum at two distinct time points (1 and 4 h), we identified key proteins involved in iron acquisition, regulation of virulence factors, and metabolic adaptations essential for evasion and resistance mechanisms. Our findings highlight potential targets for novel antimicrobial and antivirulence strategies, aimed at enhancing serum sensitivity and improving treatment outcomes against *K. pneumoniae* infections.

## Material and Methods

### Ethical Statement

The study was conducted in accordance with the Declaration of Helsinki and approved by the Brazilian National Review Board and Research Ethics Committee of the Universidade Federal do Rio Grande do Sul (CAAE #39070020.3.0000.5347). Human serum was obtained from healthy donors who agreed to participate after signing informed consent. All the methods were performed in accordance with the relevant guidelines and regulations. The sera were pooled to avoid heterogeneity among the different donors.

### Microorganism and Culture Conditions

A clinical strain of *K. pneumoniae* subsp. *pneumoniae* (ACH2), isolated from a prosthetic joint from a patient with a recurrent infection, was identified at the species level by matrix assisted laser desorption ionization time of flight (MALDI-TOF) mass spectrometry by Microflex LT (Bruker Daltonik), considering a confidence score of ≥1.7. The *in vitro* ability of extended-spectrum β-lactamase (ESBL) production was tested according to CLSI M100-S22 instructions,^13^ with *K. pneumoniae* ATCC 700603 used as standard. ESBL production was confirmed with ceftazidime (CAZ) 30 μg, CAZ–clavulanic acid (30/10 μg), cefotaxime (CTX) (30 μg), and CTX–clavulanic acid (30/10 μg).

The bacterium was cultured in BHI medium at 37 °C and 150 rpm for 16 h. After this time, cells were separated by centrifugation (11,200 × *g*, 5 min), washed three times with sterile PBS, and resuspended in 5 mL of PBS. Then, bacterial cells (OD_600_ = 0.05) were cultured in 40% native serum or heat-inactivated serum (control), whose complement system was inactivated by heat for 40 min at 56 °C.^6^ Both sera were diluted in PBS and agitated at 150 rpm and 37 °C for up to 4.5 h, as previously described.^6^

To determine if *K. pneumoniae* was able to resist human serum, samples (1 mL) were collected from each condition, and the absorbance at 600 nm was measured spectrophotometrically every 30 min.^6^ The cell viability assay was performed after 1 and 4 h of exposure to native or heat-inactivated serum (control). The samples were serially diluted, and 10 μL aliquots were drop-plated onto BHI agar plates and incubated at 37 °C overnight.

### Antimicrobial Susceptibility Testing (AST)

The strain was tested for antimicrobial susceptibility using the agar disc diffusion method on Mueller–Hinton agar (Oxoid, UK) according to the CLSI guidelines.^13^ Sixteen antibiotics were tested, representing the major classes of antimicrobial drugs for both veterinary and human medicine: ampicillin (10 μg), amoxicillin–clavulanic acid (30 μg), ceftazidime (30 μg), cefepime (30 μg), ceftriaxone (30 μg), cefuroxime (30 μg), cephalothin (30 μg), piperacillin/tazobactam (110 μg), meropenem (10 μg), ertapenem (10 μg), gentamicin (10 μg), ciprofloxacin (5 μg), and trimethoprim–sulfamethoxazole (25 μg) (all Oxoid, UK). The isolate was resistant to all 16 antibiotics tested on the basis of specific breakpoints.^13^

### Screening for Carbapenemase Production

The isolate was evaluated for carbapenemase genes by multiplex real-time polymerase chain reaction (RT-PCR) using a high-resolution melting (HRM) for *bla*_KPC_, *bla*_NDM_, *bla*_OXA-48-like_, *bla*_IMP_, *bla*_VIM_, and *bla*_GES_ as previously described.^14^ The gene *bla*_SPM_ was also evaluated in RT-PCR HRM. DNA was extracted by thermal lysis, and HRM were performed using the equipment QuantStudio 3® (Thermo Fisher).

### Sample Preparation for Mass Spectrometry

For protein extraction, bacterial cells were collected after 1 and 4 h of serum exposure, washed 3 times with PBS, and centrifuged (11,200 × *g* for 5 min each time). After that, cells were disrupted with a mortar and pestle in liquid nitrogen to a fine powder, which was collected, and 20 mM tris-HCL buffer (pH 7.5) containing protease cocktail inhibitors (Sigma, USA) was added. After centrifugation (11,200 × *g* for 10 min), the supernatants containing proteins (100 μg) were treated for analysis by mass spectrometry.^14^ Samples were diluted in digestion buffer (8 M urea and 100 mM tris-HCl pH 8.5), reduced with 5 mM tris-2-carboxyethyl-phosphine (TCEP) for 20 min at room temperature, and alkylated with 10 mM iodoacetamide at room temperature for 15 min in the dark. The proteins were digested with 2 μg of MS-grade trypsin (1:40) (Promega, USA) by incubation at 37 °C for 16 h, according to the manufacturer.

### Multidimensional Protein Identification Technology (MudPIT) Analysis

For MudPIT, peptides were pressure-loaded into a capillary packed with 2.5 cm of a strong cation exchanger (5 μm Partisphere) (Whatman, USA), followed by 2 cm of a reverse phase (3 μm Aqua C_18_) (Phenomenex, USA). The column was attached to a capillary column with a tip packed with 11 cm of C_18_ resin. The buffer solutions used were 5% acetonitrile/0.1% formic acid (buffer A), 80% acetonitrile/0.1% formic acid (buffer B), and 500 mM ammonium acetate/5% acetonitrile/0.1% formic acid (buffer C). Six steps of peptide separation (60 min each) with increasing concentrations of buffer C (0, 20, 40, 60, 80, and 100%) were performed. An additional step containing 90% buffer C and 10% buffer B was used. Three biological replicates and three technical replicates were analyzed for both *K. pneumoniae* culture conditions (native and control).

### Mass Spectrometry and Analysis of Tandem Mass Spectra

Peptides eluted from the microcapillary column were electrosprayed directly into an LTQ-Orbitrap mass spectrometer (Thermo Fisher, USA). A cycle of one full-scan spectrum (300–2000 *m/z*) followed by five data-dependent MS/MS spectra at a 35% normalized collision energy was repeated continuously throughout each step of the multidimensional separation. MS/MS spectra were analyzed using IP2 (www.integratedproteomics.com/). The search was performed using the ProLuCID algorithm against the *K. pneumoniae* subsp. *pneumoniae* HS11286 available in the NCBI database (GenBank accession: GCF_000240185.1). The peptide mass search tolerance was set to 3 Da, and carboxymethylation (+57.02146 Da) of cysteine was considered to be a static modification. ProLuCID results were assembled and filtered using the DTASelect program, applying two defined parameters (Xcorr and DeltaCN), to achieve a false-positive rate of 1%. Data are available at https://www.proteomexchange.org/, number PXD046166.

### Data Analysis

Different software programs were used to analyze the data. The software PatternLab^16^ was used to identify differentially expressed proteins: TFold module was used to select differentially expressed proteins for both conditions (native × control–heat-inactivated serum) in 1 or 4 h after serum exposure. The following parameters were used: proteins that were not detected in at least two out of three runs per condition were excluded with a *q*-value of 0.05 and a FDR of 5%. Also, an absolute fold change greater than two was used to select differentially expressed proteins (up- or downregulated). The AAPV module was used for pinpointing proteins uniquely identified under a condition using a probability of 0.01. To be included in our analysis, all proteins were required to have at least one unique peptide. The analysis was based on spectral count numbers obtained from native serum and the control exposure.

The STRING (https://string-db.org.) and Cytoscape (https://cytoscape.org/) software programs were used to construct and visualize protein interaction networks, respectively, using medium confidence (0.400) as the parameter selected. Gene Ontology categorization and KEGG analyses were performed with DAVID (https://david.ncifcrf.gov) of differentially regulated proteins dataset. All analyzed proteins met a two-fold cutoff threshold for differential expression (*p* < 0.05).

### Enzymatic Assays

To validate the proteomic findings, enzymatic assays were performed. The superoxide dismutase (SOD) assay was conducted as previously described.^15^ Briefly, a solution containing 0.05 M potassium phosphate buffer pH 7.8, 13 mM l-methionine, 75 mM NBT (nitro blue tetrazolium), 0.1 Mm EDTA, and 0.025% Triton X-100 was added to glass tubes. To start the reactions, sample and 10 Mm riboflavin was added to the tubes that were immediately placed under fluorescent light for 15 min. After this period, the absorbance was determined to be 560 nm. One SOD unit (U) was defined by NBT reduction per mL min^–1^ per mg of protein.

Furthermore, an adapted quantitative method was used to quantify siderophores production.^17^ Briefly, 1 mL of bacterial cultures exposed to native serum and control after 1 and 4 h were recovered by centrifugation (11,200 × *g*, 10 min). Supernatants (1 mL) mixed with 1 mL of CAS reagent were used to estimate siderophores. After 20 min, the mixtures were read at 630 nm on a spectrophotometer (Biospectro, Brazil). The siderophore produced was defined in percent siderophores unit (psu), which was calculated as previously described.^18^

Protein concentration was determined using the BCA kit assay (Thermo Scientific, USA).

### Serum Metabolite Assay

Based on KEGG analysis, the changes observed in protein profiles during serum exposure could lead to an accumulation of certain metabolites. To determine if specific metabolites might increase the resistance to human serum, a survival assay was performed, as previously described.^7^ Briefly, 2 mL aliquots of bacterial cells were adjusted to an OD_600_ of 1.0 with saline and centrifuged at 11,200 × *g* for 10 min at 4 °C. Then, 100 μL of native serum or control was added, together with 150 μL saline solution containing the following metabolites: 100 mM glycine,^7^ 50 mM succinate,^19^ and 10 mM thiamine.^20^ The tubes were cultured at 200 rpm for 2 h at 37 °C. A negative control containing only 250 μL of saline was used. After this time, the cells were collected by centrifugation (11,200 × *g*, 10 min at 4 °C), ressuspender in 2 mL of saline, and serially diluted (1:10) up to 10^–10^. Then, 10 μL aliquots were spread plated onto brain heart infusion (BHI) agar plates and cultured overnight at 37 °C for colony-forming unit (CFU) analysis. Dilutions yielding 20–200 CFU were used for analysis. The experiments were performed in triplicate.

### Statistical Analysis

All assays were performed in triplicate. Data generated of CFU counts, enzymatic assays, and serum metabolite assays were analyzed statistically using the Student’s *t*-test in GraphPad Prism 5 software.

## Results

### Susceptibility and Carbapenemase Genes Evaluation

The isolate presented resistance to all 16 antibiotics tested, including carbapenems, according to the AST and was positive for the *bla*_KPC_ gene by qPCR-HRM.

### *K. pneumoniae* ACH2 was Able to Resist to Human Serum

**Figure 1:**
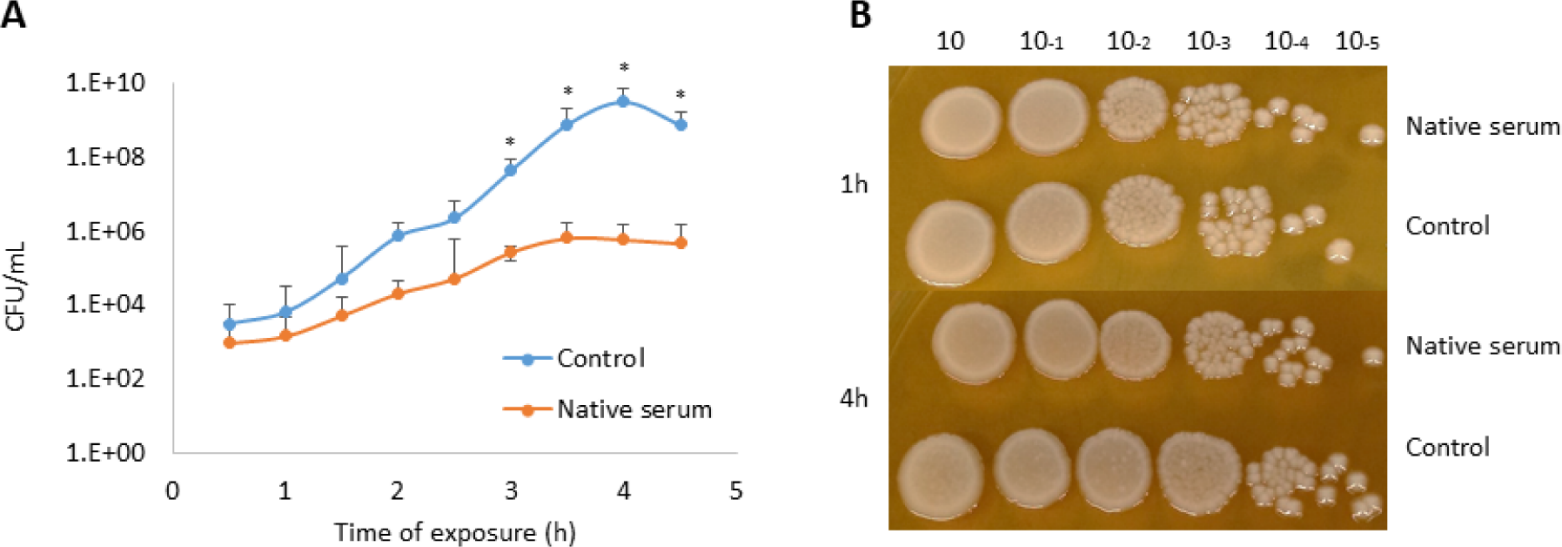
*K. pneumoniae* ACH2 is resistant to native serum. (A) The bacterium was grown in 40% native serum or control (heat-inactivated serum) for up to 4.5 h and absorbance (OD_600_) was measured. (B) Cell viability was estimated by dropping 10 μL of a serial dilution of both cultures on BHI agar plates and incubating overnight at 37 °C. Results are displayed as the mean of 3 replicates ± SEM, and significant differences are identified (*p < 0.05).

We evaluated the ability of *K. pneumoniae* ACH2 to resist and survive in the presence of human serum. The growth curve (Figure 1) indicates that there is some degree of killing when native serum was compared to control, mainly after 4 h of exposure, probably due to complement activity.^6^ However, the drop-plate assay indicates that even though a killing effect was observed, the remaining cells were able to resist and survive in the presence of serum (Figure 1B).

### Overview of the Proteomic Analysis

**Figure 2:**
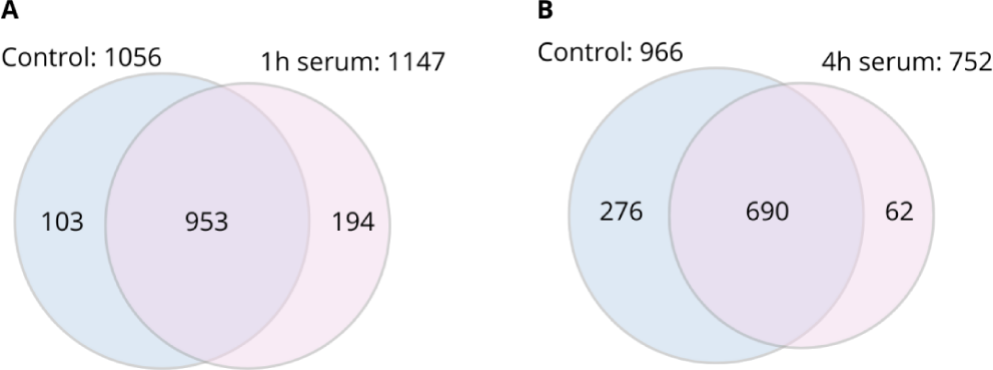
Distribution of *K. pneumoniae* ACH2 proteins identified after serum exposure. Venn diagrams show the dispersion of proteins identified after (A) 1 and (B) 4 h of serum exposure, when comparing control (heat-inactivated serum) against native serum. Blue circle: number of unique proteins identified after exposure to the control; pink circle: number of unique proteins identified after exposure to serum. Intersection of circles indicates proteins common to both conditions.

The MudPIT analysis identified a total of 1250 proteins after 1 h of serum exposure (Figure 2A). Among the 953 proteins identified for both conditions, 6 were identified as downregulated and 5 as upregulated when native serum was compared with the control (Table 1). The most downregulated protein was ecotin (12.5-fold), a protein involved in host immune modulation, while the most upregulated was 30S ribosomal protein S4 (43.33 fold), related to ribosome assembly. In addition, 194 proteins (15.5%) were identified as unique or exclusive of *K. pneumoniae* exposed to native serum, when compared with the control condition (Table S1).

**Table 1:** Proteins Differentially Expressed in 1 and 4 h after Serum Exposure^a^

Accession number^b^	Fold change^c^	*p* value	Protein description
1 h after serum exposure			
AEW62391.1	–12.50	0.043	ecotin
AEW59072.1	–12.00	0.020	aspartate ammonia-lyase
AEW61996.1	–10.20	0.017	putative formate acetyltransferase
AEW59229.1	–8.17	0.013	putative sugar phosphate isomerase/epimerase
AEW63605.1	–6.50	0.004	tryptophanyl-tRNA synthetase
AEW63102.1	–5.85	0.0007	glycine dehydrogenase
AEW59696.1	4.83	0.002	acetoin:2,6-dichlorophenolindophenol oxidoreductase, alpha subunit
AEW58725.1	5.93	0.003	glutamine synthetase
AEW63549.1	16.79	0.006	30S ribosomal protein S5
AEW62607.1	17.40	0.009	inosine-5′-monophosphate dehydrogenase
AEW63543.1	43.33	0.026	30S ribosomal protein S4
4 h of serum exposure			
AEW60188.1	–70.67	0.025	hypothetical protein KPHS_14900
AEW61996.1	–68.00	0.010	putative formate acetyltransferase
AEW63713.1	–55.13	0.048	universal stress protein A
AEW63102.1	–38.00	7.23 × 10^–08^	glycine dehydrogenase
AEW62480.1	–29.00	0.014	3-oxoacyl-(acyl carrier protein) synthase I
AEW63351.1	–27.08	0.023	uronate isomerase
AEW62331.1	–23.29	0.0129	D-lactate dehydrogenase
AEW61674.1	–21.73	0.004	superoxide dismutase (Fe)
AEW59900.1	–19.60	0.018	adenylate kinase
AEW63814.1	–16.00	0.041	phosphoglyceromutase
AEW63325.1	–15.46	0.017	dihydroxyacetone kinase subunit DhaK
AEW61664.1	–13.75	0.018	superoxide dismutase (Cu/Zn)
AEW60009.1	–13.54	0.0008	lysyl-tRNA synthetase
AEW62565.1	–12.56	0.022	malic enzyme
AEW59130.1	–12.00	0.005	putative l-ascorbate 6-phosphate lactonase
AEW60007.1	–11.88	0.018	lysine decarboxylase 1
AEW59072.1	–11.45	0.021	aspartate ammonia-lyase
AEW63084.1	–11.41	0.006	lysyl-tRNA synthetase
AEW61506.1	–10.76	0.031	putative oxidoreductase
AEW59139.1	–10.18	0.047	30S ribosomal protein S6
AEW60509.1	–10.04	0.009	formate acetyltransferase 1
AEW61062.1	–8.17	0.006	phenylacetaldehyde dehydrogenase
AEW60777.1	–7.63	0.013	hypothetical protein KPHS_20790
AEW59390.1	–7.00	0.013	phosphopentomutase
AEW60479.1	–6.91	0.002	pyruvate dehydrogenase
AEW59422.1	–6.15	0.004	molecular chaperone DnaK
AEW58896.1	–5.73	0.003	50S ribosomal protein L1
AEW59614.1	–5.73	0.002	30S ribosomal protein S2
AEW63118.1	–5.10	0.001	fructose-bisphosphate aldolase
AEW58725.1	7.22	0.005	glutamine synthetase
AEW59623.1	9.63	0.002	chaperone protein Skp

a Statistically differentially expressed proteins were identified using Patternlab’s TFold module, with an absolute fold change greater than 2.0 (BH-FDR 0.05).

b According to database GenBank accession: GCF_000240185.1.

c Based on spectral count numbers obtained from native serum and control exposure. Negative numbers represent downregulated proteins in native serum when compared to control (heat-inactivated serum), and positive numbers represent upregulated proteins in native vs control.

In the same way, the MudPIT analysis after 4 h of serum exposure identified a total of 1028 proteins (Figure 2B), of which 690 proteins were identified for both conditions. Among these, 29 proteins were identified as downregulated and 2 as upregulated. Also, 62 (6.03%) were identified as unique or exclusive to native serum, when compared to the control condition (heat inactivated serum; Table S2). We identified a range of proteins across the three replicates: 723–1241 for control, 818–1363 for 1 h, and 678–1578 for 4 h of serum exposure.

### Gene Ontology (GO) Analysis of the Differentially Expressed Proteins

**Figure 3:**
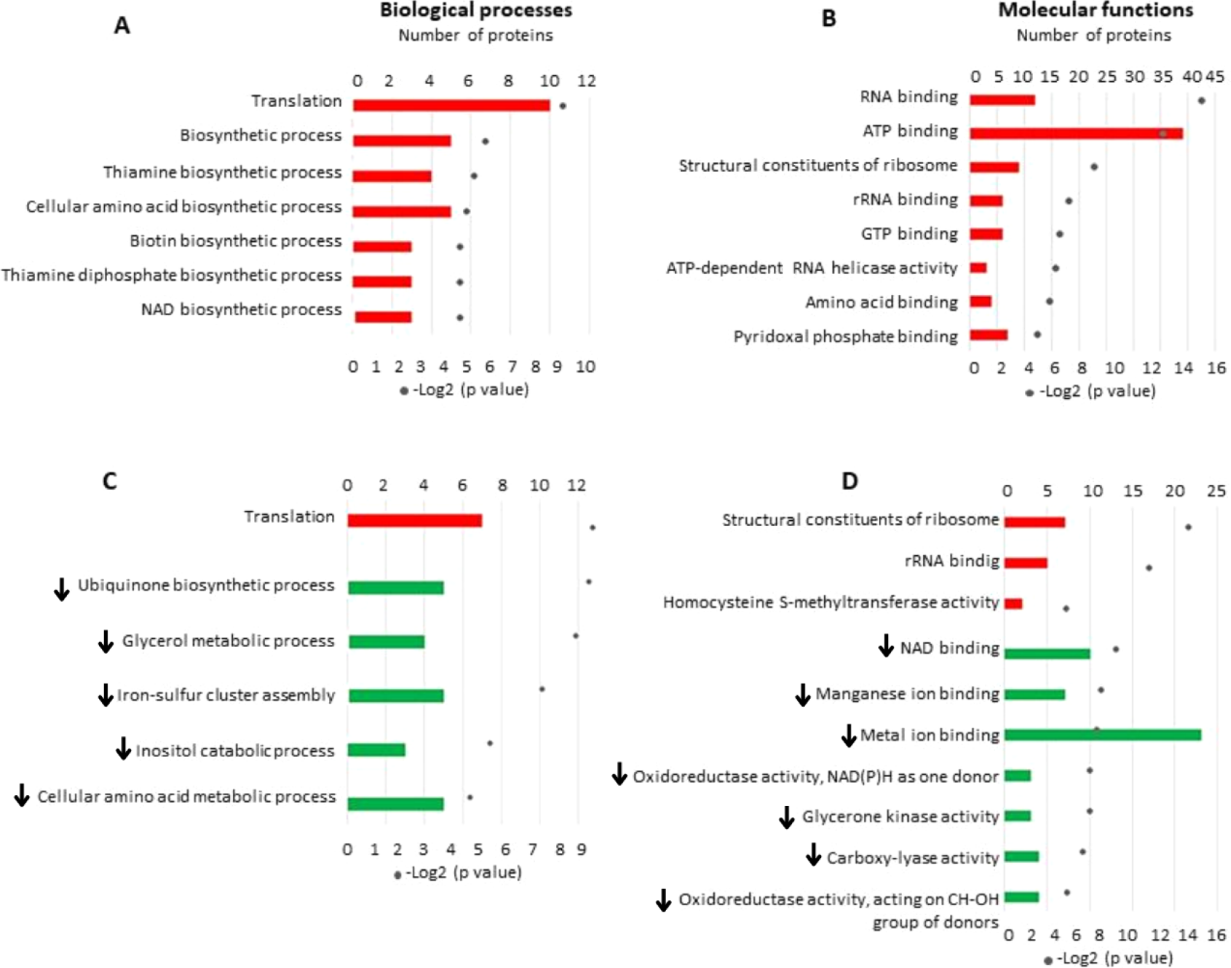
Plot of Gene Ontology classification of all differentially expressed proteins from *K. pneumoniae* ACH2 when comparing native vs control serum (heat-inactivated) after 1 and 4 h of exposure. Biological process classification for (A) 1 h or (C) 4 h; Molecular function classification for (B) 1 h or (D) 4 h; All tabulated proteins met a two-fold cutoff threshold for differential expression (*p* < 0.05). Red bars: upregulated proteins; green bars: downregulated proteins (with arrows).

All differentially expressed proteins were subjected to GO analysis and classified into biological processes and molecular functions (Figure 3). After 1 h, proteins related to translation and biosynthetic processes were identified with higher significant difference (*p* > 0.05) in biological processes classification (Figure 3A). After 4 h, translation was upregulated, while metabolic and biosynthetic processes of ubiquinone, glycerol, amino acids, inositol, and iron–sulfur cluster assembly were down-regulated (Figure 3C). For molecular functions, a higher number of upregulated proteins were related to ATP binding after 1 h (Figure 3B), and proteins related to ribosome and metal ion binding identified as up- and downregulated after 4 h of serum exposure, respectively (Figure 3D).

### Protein Network Analysis

**Figure 4:**
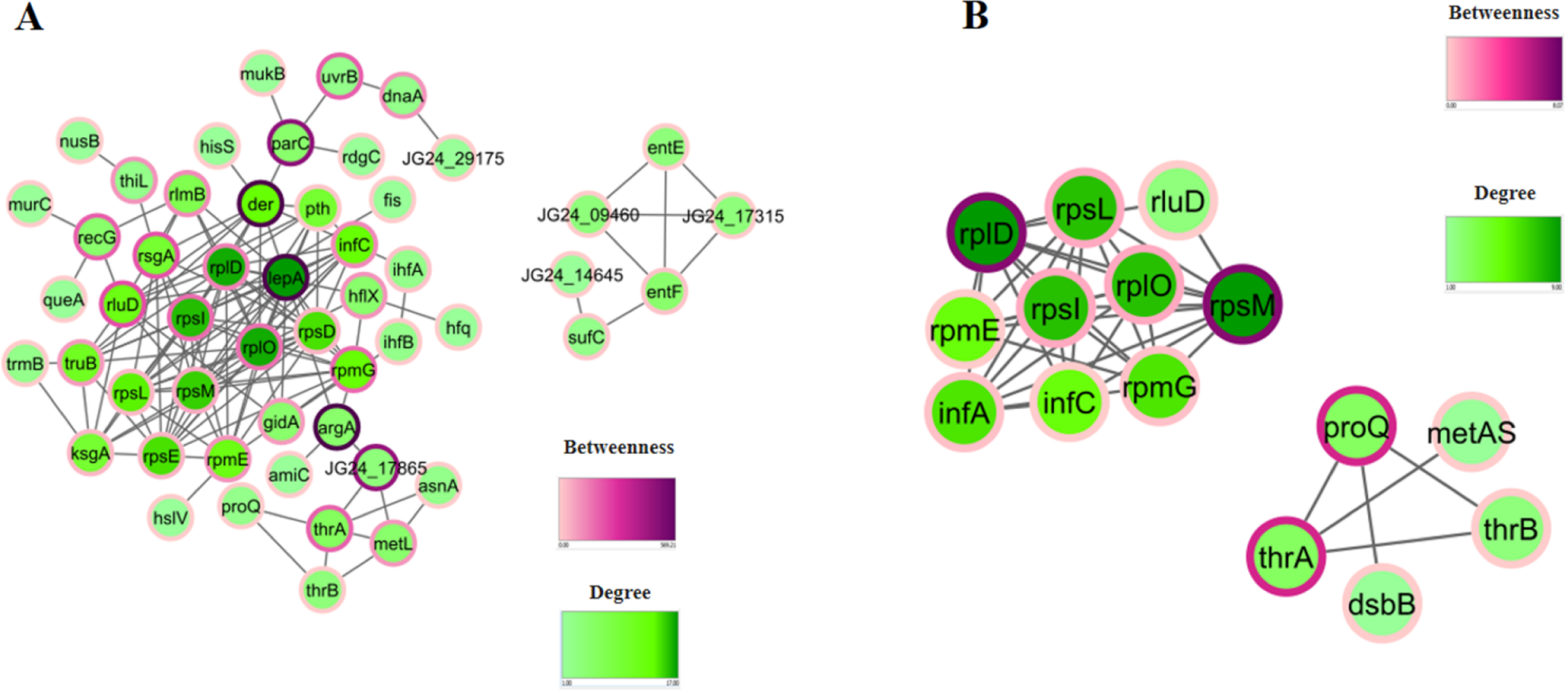
Interactome analysis of *K. pneumoniae* ACH2 proteins identified after (A) 1 h or (B) 4 h of serum exposure. The STRING database was used to determine the protein network analysis of proteins identified for both time points evaluated (*p* < 0.05). Betweenness measures the level of interaction between distant proteins, and degree measures the number of connections of a protein in a network.

To evaluate the protein network analysis, we performed a network analysis for all differentially regulated (up, down, and exclusive) proteins. We identified after 1 h two clusters, one containing proteins related to ribosomes (*n* = 14) and another with iron-associated proteins: EntE (enterobactin peptide non-ribosomal synthetase EntE) and EntF (enterobactin peptide non-ribosomal synthetase EntF) (Figure 4A). The first cluster encompasses LepA as a protein with the highest degree and betweeness. In addition, Der and Hfq proteins, responsible for the regulation of virulence factors, were presented in this cluster.

After 4 h, two clusters were identified: one containing 10 proteins related to ribosomes and another cluster containing proteins related to chaperones and amino acid biosynthesis: ProQ (RNA chaperone ProQ), DsbB (disulfide bond formation protein B), ThrA (bifunctional aspartokinase/homoserine dehydrogenase), ThrB (homoserine kinase), and MetAS (homoserine O-succinyltransferase) (Figure 4B). No protein network was identified for downregulated proteins after 1 or 4 h after serum exposure. Betweenness measures the level of interaction between distant proteins, and degree measures the number of connections of a protein in a network.

### Validation of Proteomic Data

**Figure 5:**
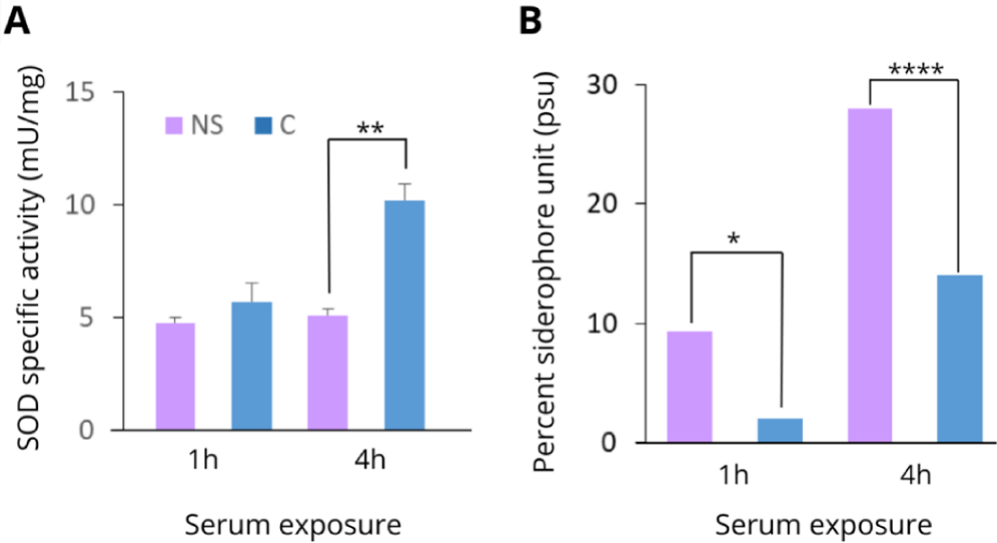
Specific activities of (A) superoxide dismutase and (B) siderophore production of *K. pneumoniae* ACH2 exposed to native serum for 1 and 4 h. Results are displayed as the mean of 3 replicates ± SEM, and significant differences are identified (*p < 0.05; **p < 0.01; ****p < 0.0001).

To validate the proteomic data identified, SOD activity was quantified by an enzymatic assay. We identified Fe-SOD and Cu/Zn-SOD as downregulated (−21.73 and −13.75 times, respectively) after 4 h of serum exposure. SOD activity was lower in native serum than in the control, corroborating the proteomic data (Figure 5A).

Since enterobactin and other proteins related to siderophore production (EntE and EntF) were identified as upregulated 1 h after serum exposure, we performed a siderophore production assay. In agreement with our proteomic findings, siderophore production was higher in native serum than in the control at both evaluated time points (Figure 5B).

### Metabolic Pathways Impacted by Serum Exposure

**Figure 6:**
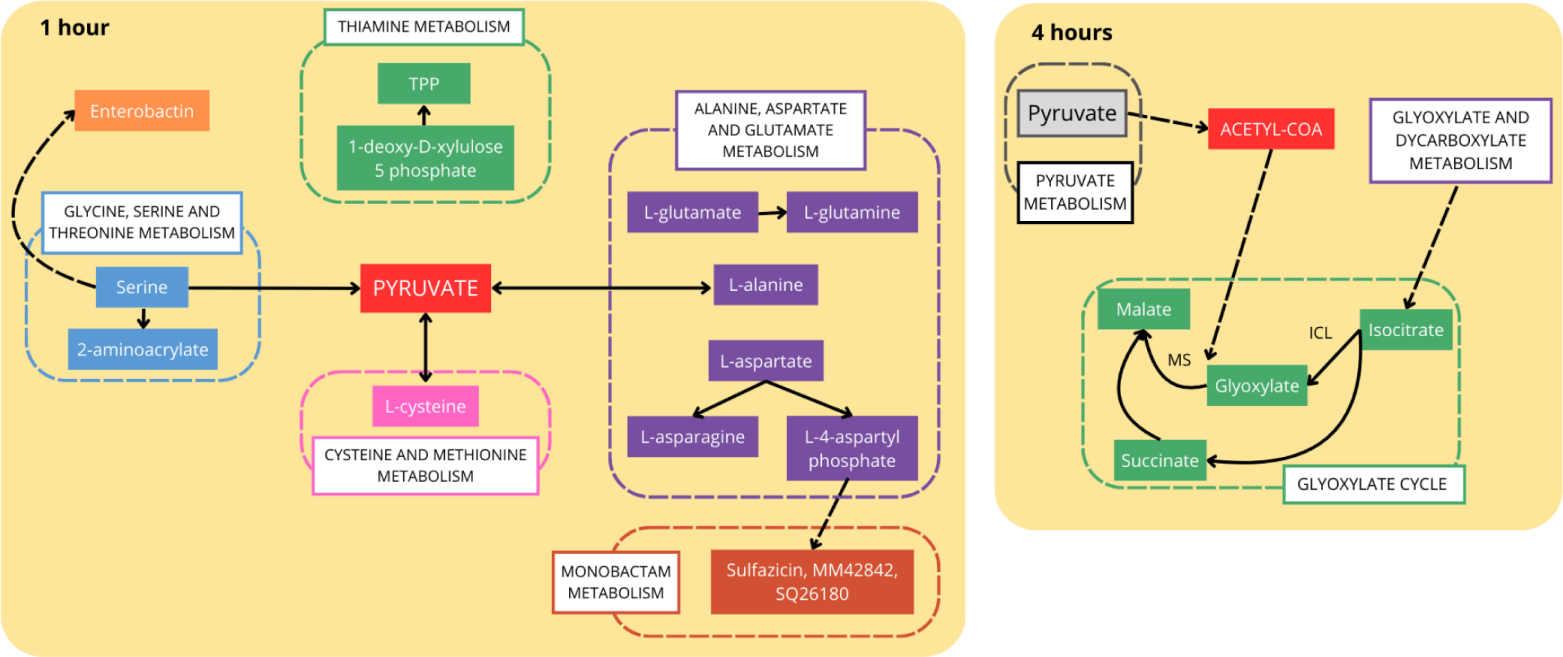
Overview of metabolic pathways impacted in *K. pneumoniae* ACH2 after 1 and 4 h of serum exposure. TPP: thiamine pyrophosphate. ICL: isocitrate lyase; MS: malate synthase. Full line: direct production; dashed line: indirect production. Figure was generated based on KEGG results.

It has been proposed that successful evasion of the immune system by pathogenic bacteria is related to a proper metabolic adaptation to human serum.^6^ For this reason, we evaluated the metabolic changes caused by human serum exposure in *K. pneumoniae* ACH2. According to DAVID analysis, six metabolic pathways were impacted after 1 h: four related to amino acid metabolism (glycine, serine, and threonine; thiamine; alanine, aspartate, and glutamate; and cysteine and methionine metabolism), biosynthesis of siderophore, and monobactam biosynthesis. When these metabolic changes are evaluated, pyruvate seems to play a central role during the first hour of serum exposure (Figure 6). Serine can be converted to the metabolite 2-aminoacrylate, which is involved in both stress signaling and the production of enterobactin, a siderophore also identified in our analysis. In addition, thiamine pyrophosphate (TPP), l-asparagine, and l-glutamine, which function in the maintenance of cellular metabolism, could accumulate (Figure 6).

After 4 h of serum exposure, six pathways were impacted: glycerolipid metabolism, glycerophospholipid metabolism, pyruvate metabolism, glyoxylate and dicarboxylate metabolism, fatty acid degradation, and biosynthesis of ubiquinone and other terpenoid-quinones, which is consistent with recent report.^5^ Analyzing these pathways, it seems pyruvate is still produced, generating acetyl-CoA, which is used in the glyoxylate cycle (GC). This cycle seems to be induced by human serum, since isocitrate lyase (ICL) and malate synthase (MS), key enzymes, were exclusively identified in serum, even after 1 h (Figure 6). In addition, isocitrate is produced by glyoxylate and dicarboxylate metabolism, feeding the GC cycle.

### Serum–Metabolite Assays

**Figure 7:**
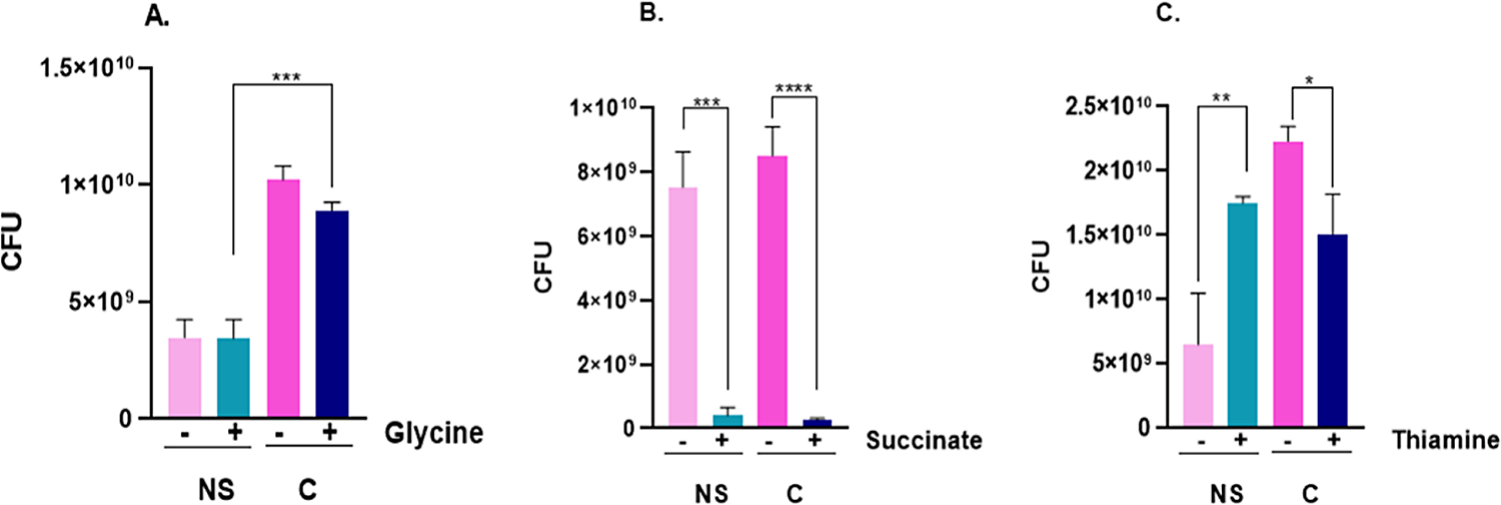
Effect of metabolites upon *K. pneumoniae* ACH2 serum resistance. Colony forming unit (CFU) number of *K. pneumoniae* ACH2 cells incubated with 100 μL native serum in the presence or absence of (A) 100 mM glycine, (B) 50 mM succinate, or (C) 10 mM thiamine. Results are displayed as the mean of 3 replicates ± SEM, and significant differences are identified (*p < 0.05, **p < 0.01, ***p < 0.001, ****p < 0.0001). NS = native serum; C = control (heat-inactivated serum).

To assess whether specific metabolites can enhance resistance to human serum, we conducted three independent experiments using glycine, succinate, and thiamine (Figure 7). These metabolites were chosen because glycine has been shown to decrease serum resistance in *E. coli*,^7^ thiamine might increase the production of TPP, increasing serum resistance, and succinate is a by-product of the GC.

Exogenous glycine did not induce serum resistance, measured in colony-forming units. However, we observed a significant difference when comparing native serum versus the control with this metabolite (Figure 7A). For succinate addition, a pronounced decrease in cell viability for both native serum and the control was observed (Figure 7B). The only metabolite tested that induced resistance to native serum was thiamine (Figure 7C).

**Figure 8:**
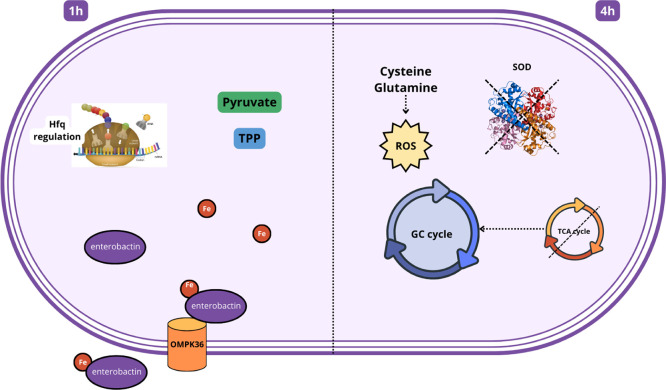
Overview of the proteomic analysis of *K. pneumoniae* ACH2 exposed to native human serum. In 1 h: secretion of enterobactin to acquire iron from native serum; enterobactin transpose cell wall through pores formed by OMPK36. Metabolites such as pyruvate and thiamine pyrophosphate (TPP) are being produced and related to virulence and bacterial survival. There is also the production of proteins that regulate protein expression and synthesis, such as Hfq (via ribosome regulation). In 4 h: metabolic shift induces glyoxylate cycle (GC) instead TCA cycle. Superoxide dismutase (SOD) is inhibited, as well as other stress response. Cysteine and glutamine might act as detecting ROS, boosting oxidative response, inducing serum resistance.

Based on the data identified in this work, an overview of cellular alterations probably associated with bacterial resistance to human serum was created (Figure 8), and relevant metabolites and metabolic reprogramming identified by proteomics were highlighted.

## Discussion

Serum resistance and immune system evasion are known mechanisms of several pathogenic microorganisms that use different strategies to subvert host immune responses, such as molecular mimicry and virulence factor production, interfering with host defense.^21^ However, the molecular mechanisms are still far from being elucidated.^7^ Like other microorganisms, *K. pneumoniae* has developed strategies to suppress the host immune system and create a suitable environment for its own survival, causing systemic infections.^22^ Here, using a proteomic approach, we identified metabolic changes and other proteins that might possibly be involved in serum survival, shedding light on the possible molecular mechanisms involved in this strategy.

### *K. pneumoniae* is Able to Resist Human Serum

The strain used in this work was collected from a patient with recurrent infections associated with a prosthetic device. As previously reported, *K. pneumoniae* can be found in the intestinal microbiota, where it may transverse the intestinal epithelium, resulting in systemic infections.^22^ As expected, this strain was able to proliferate in human serum, a phenomenon that has already been observed for several bacteria, including extraintestinal *E. coli*.^6^ Since heat-inactivated serum is widely used for the denaturation of complement proteins, the first line of defense against pathogens,^23^ without changing the nutrient composition,^6^ it was used as a control.

### Proteomic Profile of *K. pneumoniae* Exposed to Serum

When a pathogen invades its host, it must overcome the molecular barriers. Like other microorganisms, *K. pneumoniae* employs various mechanisms and gene sets to avoid complement attack. Rapid post-transcriptional control and translation enable immune evasion and resistance, influencing infection success. This study evaluated differentially expressed proteins after 1 and 4 h of serum exposure in order to compare the protein profiles at both time points.

In the first hour of serum exposure, *K. pneumoniae* induced protein expression related to translation and its control, which is crucial for responding rapidly to the serum environment. The most upregulated protein identified was 30S ribosomal protein S4 (RpsD), which, along with S5 (RpsE) (also upregulated), supports translational accuracy and antibiotic resistance.^24^ Ribosomal proteins may also influence virulence and stress response in pathogens.^25^ The most downregulated protein identified after 1 h of serum exposure was ecotin, a serine protease inhibitor, critical for serum evasion.^26^ Despite the key function of ecotin in serum evasion, probably *K. pneumoniae* uses other mechanisms to traverse this barrier, as potentially identified here. Notably, Skp, which is the most up-regulated protein at the 4 h, plays a key role in the biogenesis of outer membrane proteins (OMPs). Skp has been associated with several important bacterial functions, including adhesion, immune evasion, virulence, and antibiotic resistance.^27^

### Interactome Network

Hub proteins were identified in a network, including elongation factor 4 (LepA), crucial for protein quality control and stress response, indirectly modulated antimicrobial entry through the membrane.^28^ Der protein and Hfq played key roles in regulating bacterial adhesion, invasion, and virulence,^29−32^ with Hfq being a promising target for new antimicrobial strategies.^30^ A cluster of proteins related to enterobactin (EntE and EntF), which are crucial for iron acquisition, was also found. Recent studies suggest that *K. pneumoniae* siderophore contribute to inflammation and bacterial spread in pneumonia, impacting survival and resistance through enhanced virulence.^1,33−35^

After 4 h of serum exposure, translation remained upregulated. Proteins like glutamine synthetase, contributing to nitrogen metabolism, was upregulated. Glutamine, serving as an environmental indicator, may induce virulence gene expression in bacteria,^36^ suggesting an internal response (1 h) to induce virulence genes (4 h). ABC transporters were identified, indicating nutrient adaptation and invasion, as previously observed in *Moraxella catarrhalis*.^37^ Interactome analysis at 4 h revealed clusters involving ribosomes and proteins such as ProQ, ThrA, ThrB, MetAS, and DsbB. ProQ, an RNA chaperone, modulates virulence factors synthesis and host immune pathways in *S. typhimurium*,^38^ while DsbA/DsbB form disulfide bonds in bacterial virulence factors, representing potential targets for antibacterial compounds.^39^ These proteins were associated with amino acid biosynthesis, disulfide bond formation, and virulence. In summary, *K. pneumoniae* responded to 1 h of serum exposure with rapid translation, siderophore production, and virulence regulation, with Hfq playing a key role. At 4 h, translation continued alongside amino acid biosynthesis and disulfide bond formation.

During infection, immune cells release toxic superoxide and other reactive oxygen species (ROS), with superoxide dismutases (SOD) considered to be virulence factors in bacteria. Our analysis indicated reduced levels of stress-related proteins (Fe and Cu/Zn SOD, oxidoreductases, and USPA) with confirmed lower SOD activity after 4 h. Notably, *K. pneumoniae* lacking Fe- and Cu/Zn-SOD promoted acetate formation, potentially enhancing survival via the glyoxylate cycle (GC),^40^ consistent with our findings. Loss of specific USP (UspA616) in *Micrococcus luteus* led to increased expression of malate synthase and isocitrate lyase, key enzymes of the GC,^41^ suggesting stress protein deregulation could promote bacterial stress tolerance through metabolic shifts between the TCA cycle and the GC.

### Serum Exposure Causes Metabolic Shifting in *K. pneumoniae*

At one hour of serum exposure, pyruvate production via multiple pathways is pivotal in metabolism and impacts virulence, oxidative stress resistance, survival, and capsule biosynthesis in different bacteria.^5,42,43^ In addition, thiamine metabolism was up-regulated, resulting in thiamine pyrophosphate (TPP) production, essential for various cellular processes, including pathogenesis in *P. aeruginosa*(44) and defense mechanisms in *E. coli*.^20^ Additionally, TPP-dependent pyruvate complexes aid in iron acquisition,^45^ consistent with our findings. Exogenous thiamine supplementation in serum enhanced resistance, highlighting thiamine biosynthesis proteins as potential targets due to their absence in human cells.^44^

Early serum exposure induced specific amino acid metabolism pathways, leading to the accumulation of asparagines, glutamines, and cysteine. Glutamine might influences virulence gene expression,^36^ while asparagine is linked to virulence in *Salmonella* spp.^46^ In a variety of pathogenic and nonpathogenic bacteria, cysteine has been associated with the ability to resist oxidative stress, including virulence and antibiotic resistance.^47^ These amino acids may enhance serum resistance by inducing virulence genes and increasing oxidative stress resistance.

After four hours of serum exposure, glycerolipid metabolism pathways crucial for bacterial membrane integrity^48^ were regulated. The GC, known for its roles in stress defense, host infection, and resistance to antibiotic in several pathogenic microrganisms,^49,50^ was also activated. The activation of the GC, along with other metabolites like cysteine and pyruvate, may explain the observed downregulation of oxidative stress-related proteins. Studies with a mutant lacking malate synthase (a GC enzyme) in *M. tuberculosis* showed reduced stress tolerance and survival in macrophages.^51^ Addition of exogenous succinate, a GC by-product intensifying bacterial virulence, unexpectedly decreased cell viability, possibly due to inducing catabolic repression of the GC. This promoted the tricarboxylic acid (TCA) cycle via substrate activation, increasing NADH production, and rendering higher sensitivity to antibiotics and serum.^7^ Exposing *E. coli* to serum with exogenous glycine similarly increased NADH production via the TCA cycle, reducing serum resistance.^7^ In our analysis, exogenous glycine did not induce serum sensitivity, possibly due to GC activation rather than the involvement of the TCA cycle in *K. pneumoniae*. Thus, the GC appears crucial during serum exposure, supporting cell maintenance and stress response. Disrupting this cycle may restore serum sensitivity. Evaluation of succinate in *in vivo* infection models could further assess its efficacy, with GC proteins being potential novel targets for antimicrobial therapies given their absence in human cells.

In conclusion, *K. pneumoniae* ACH2 has developed mechanisms of metabolic reprogramming likely associated with immune evasion and resistance. Our findings suggest that this bacterium induces the expression of virulence genes, enhances translation, and alters its metabolism to survive and evade the host immune system. Iron acquisition through enterobactin and pyruvate metabolism is central to nutrient homeostasis, and exogenous succinate decreasing cell viability. We have identified key proteins involved in gene expression regulation, translation, and metabolic reprogramming that may contribute to serum resistance. Nevertheless, further experiments are needed to fully explore the intricate molecular landscape described here in relation to serum resistance and to develop novel therapeutic targets.

## Supporting Information Available

Exclusive proteins after 1 h exposure; exculsive proteins after 4 h exposure (PDF)
