# Supplementary material for: Metabolic Reprogramming of Klebsiella pneumoniae Exposed to Serum and Its Potential Implications in Host Immune System Evasion and Resistance
Source: J Proteome Res. 2024 Oct 3;23(11):4896–906. doi: 10.1021/acs.jproteome.4c00286 (PMC11536433; doi:10.1021/acs.jproteome.4c00286)
Supplement: Supplementary file 1 — pr4c00286_si_001.pdf [file pr4c00286_si_001.pdf]

Metabolic reprogramming of *Klebsiella pneumoniae* exposed to serum and its potential implications in host immune system evasion and resistance

Amanda Naiara Silva Moraes[1], Juliana Miranda Tatara[1], Rafael Lopes da Rosa[1], Franciele Maboni Siqueira[2], Guilherme Domingues[3], Markus Berger[4,5], Jorge Almeida Guimarães[4], Afonso Luís Barth[6], Patricia Orlandi Barth[6], John R. Yates III[7], Walter Orlando Beys-da-Silva[1,8], Lucélia Santi[1,8,*]

[1] Post-Graduation Program in Cellular and Molecular Biology, Federal University of Rio Grande do Sul. Porto Alegre, RS. Brazil. 91501-970

[2] Faculty of Veterinary, Federal University of Rio Grande do Sul. Porto Alegre, RS. Brazil. 91540-000

[3] Bruno Born Hospital. Lajeado, RS. Brazil. 95900-010

[4] Center of Experimental Research, Clinical Hospital of Porto Alegre. Porto Alegre, RS. Brazil. 90035-903

[5] Tick-Pathogen Transmission Unit, Laboratory of Bacteriology, National Institute of Allergy and Infectious Diseases, Hamilton, MT, USA. 20892

[6] Bacterial Resistance Research Laboratory, Clinical Hospital of Porto Alegre. Porto Alegre, RS. Brazil. 90035-903

[7] Department of Molecular Medicine, Scripps Research. La Jolla, CA. USA. 92037

[8] Faculty of Pharmacy, Federal University of Rio Grande do Sul. Porto Alegre, RS. Brazil. 90610-000

Table of contents:

Supplementary Table 1

Supplementary Table 2

Supplementary Table 1. Exclusive proteins identified after 1 hour of native serum exposure. Proteins were identified using the software Patternlab, module AAPV, using a probability 0.01.

Accession number *	Spectral count	Protein identification
AEW63575.1	242	30S ribosomal subunit protein S12
AEW60145.1	124	enterobactin synthase subunit E
AEW60659.1	62	ribonuclease E
AEW63547.1	51	50S ribosomal protein L15
AEW62901.1	50	GDP/GTP pyrophosphokinase
AEW63488.1	48	regulator of ftsI, penicillin binding protein 3, septation function
AEW63405.1	40	ATP-dependent RNA helicase DeaD
AEW59216.1	38	leucyl aminopeptidase
AEW59510.1	32	UDP-N-acetylmuramate--L-alanine ligase
AEW63837.1	28	50S ribosomal protein L33
AEW63565.1	27	50S ribosomal protein L4
AEW60915.1	27	DNA-binding transcriptional regulator TyrR
AEW58928.1	25	isocitrate lyase
AEW62887.1	24	sulfate adenylyltransferase subunit 2
AEW60369.1	23	glutamine ABC transporter permease protein
AEW62597.1	22	polyphosphate kinase
AEW61822.1	22	cysteine desulfurase ATPase componente
AEW60144.1	20	isochorismate synthase
AEW59491.1	18	isopropylmalate isomerase large subunit
AEW58701.1	17	asparagine synthetase AsnA
AEW60181.1	17	putative aminotransferase
AEW63797.1	17	putative glutathione S-transferase
AEW60138.1	16	ATP-dependent serine activating enzyme
AEW62611.1	16	GTP-binding protein EngA
AEW59410.1	16	bifunctional aspartokinase I/homoserine dehydrogenase I
AEW59458.1	15	dimethyladenosine transferase
AEW58826.1	15	ATP-dependent RNA helicase RhlB
AEW62669.1	15	L-aspartate oxidase
AEW59810.1	14	1-deoxy-D-xylulose-5-phosphate synthase
AEW63776.1	14	major cold shock protein
AEW63769.1	14	putative sugar phosphate isomerase/epimerase
AEW58785.1	13	bifunctional aspartate kinase II/homoserine dehydrogenase II
AEW58930.1	13	transcriptional repressor IclR
AEW62987.1	13	N-acetylglutamate synthase
AEW63426.1	12	23S rRNA methyltransferase J
AEW92118.1	12	fertility inhibition protein (plasmid)
AEW59469.1	12	ATP-dependent helicase HepA
AEW61883.1	12	dsDNA-mimic protein
AEW62915.1	12	L-serine deaminase 2

AEW62677.1	12	putative NAD(P)-binding and ATP-binding acyl-CoA synthetase
AEW58980.1	12	excinuclease ABC subunit A
AEW62861.1	11	S-adenosylmethionine-dependent methyltransferase
AEW62256.1	11	dTDP-D-glucose 4,6-dehydratase
AEW61020.1	11	Oxidoreductase
AEW59115.1	11	putative GTPase HflX
AEW58916.1	11	hypothetical protein KPHS_02180
AEW59112.1	11	DNA mismatch repair protein
AEW58821.1	11	ATP-dependent DNA helicase Rep
AEW63862.1	11	bifunctional (p)ppGpp synthetase II/ guanosine-3',5'-bis pyrophosphate 3'-pyrophosphohydrolase
AEW61325.1	10	6-phosphogluconate dehydrogenase
AEW62851.1	9	iron ABC superfamily ATP binding cassette transporter, binding protein
AEW60206.1	9	hypothetical protein KPHS_15080
AEW62614.1	9	histidyl-tRNA synthetase
AEW59571.1	9	sugar fermentation stimulation protein A
AEW60760.1	9	putative glycosyl transferase
AEW62399.1	9	OmpK36 porin
AEW58782.1	8	50S ribosomal protein L31
AEW60847.1	8	putative hydroxylase
AEW60146.1	8	2,3-dihydro-2,3-dihydroxybenzoate synthetase, isochroismatase
AEW62446.1	8	aminotransferase AlaT
AEW63545.1	8	30S ribosomal protein S13
AEW62598.1	8	Exopolyphosphatase
AEW58927.1	8	malate synthase
AEW61548.1	8	cryptic nitrate reductase 2 alpha subunit
AEW63718.1	8	hypothetical protein KPHS_50200
AEW63359.1	7	hypothetical protein KPHS_46610
AEW60517.1	7	integration host factor subunit beta
AEW62671.1	7	ATP-dependent RNA helicase SrmB
AEW59924.1	7	phosphoribosylaminoimidazole carboxylase ATPase subunit
AEW62886.1	7	sulfate adenylyltransferase subunit 1
AEW61342.1	7	deoxyribose-phosphate aldolase
AEW62634.1	7	inositol monophosphatase
AEW58816.1	7	threonine dehydratase
AEW63765.1	7	biotin sulfoxide reductase
AEW58971.1	7	tRNA-dihydrouridine synthase A
AEW59114.1	6	RNA-binding protein Hfq
AEW58942.1	6	hypothetical protein KPHS_02440
AEW62674.1	6	putative methyltransferase
AEW61628.1	6	alcohol dehydrogenase class III
AEW63467.1	6	30S ribosomal protein S9
AEW60556.1	6	ABC transport system ATP-binding component
AEW60201.1	6	D-alanyl-D-alanine carboxypeptidase DacA

AEW63840.1	6	bifunctional phosphopantothenoylcysteine decarboxylase/phosphopantothenate synthase
AEW62643.1	6	putative NAGC-like transcriptional regulator
AEW60135.1	6	ferrienterobactin receptor
AEW61659.1	5	transcriptional regulator SlyA
AEW63160.1	5	tRNA (guanine-N(7))-methyltransferase
AEW61505.1	5	hypothetical protein KPHS_28070
AEW62318.1	5	putative ATP-binding protein
AEW60143.1	5	iron-enterobactin transporter periplasmic binding protein
AEW63998.1	5	phosphate transporter subunit
AEW60575.1	5	Acylphosphatase
AEW60136.1	5	enterochelin esterase
AEW58910.1	5	thiamine-phosphate pyrophosphorylase
AEW61934.1	5	4-diphosphocytidyl-2-C-methyl-D-erythritol kinase
AEW62685.1	5	23S rRNA pseudouridine synthase D
AEW63681.1	5	cell division protein FtsE
AEW58777.1	5	ATP-dependent protease peptidase subunit
AEW63976.1	5	chromosomal replication initiator protein DnaA
AEW63832.1	5	putative glycosyltransferase
AEW59142.1	5	putative cell envelope opacity-associated protein A
AEW63410.1	5	tRNA pseudouridine synthase B
AEW58752.1	5	repressor of galETK operon
AEW60530.1	5	condesin subunit B
AEW59642.1	5	DL-methionine transporter substrate-binding subunit
AEW63262.1	5	DNA topoisomerase IV subunit A
AEW62862.1	4	hemin ABC superfamily ATP binding cassette transporter, binding protein HmuT
AEW63500.1	4	DNA-binding protein Fis
AEW61184.1	4	dithiobiotin synthetase
AEW61869.1	4	hypothetical protein KPHS_31710
AEW59124.1	4	23S rRNA (guanosine-2'-O-)-methyltransferase
AEW62986.1	4	N-acetylmuramoyl-L-alanine amidase
AEW59498.1	4	LacI family transcriptional regulator, fructose operon transcriptional repressor
AEW59378.1	4	16S ribosomal RNA m2G1207 methyltransferase
AEW59772.1	4	recombination associated protein
AEW62777.1	4	ribonucleotide-diphosphate reductase alpha subunit
AEW60170.1	4	hypothetical protein KPHS_14720
AEW63283.1	4	bifunctional heptose 7-phosphate kinase/heptose 1-phosphate adenyltransferase
AEW58911.1	4	thiamin biosynthesis protein for pyrimidine moiety
AEW60383.1	4	putative transport system ATP-binding component
AEW61853.1	4	phospho-2-dehydro-3-deoxyheptonate aldolase
AEW58716.1	4	serine/threonine protein kinase RdoA
AEW63864.1	4	ATP-dependent DNA helicase RecG

AEW59411.1	4	homoserine kinase
AEW62664.1	4	GTP-binding protein LepA
AEW62355.1	4	ferric iron-catecholate outer membrane transporter
AEW60544.1	4	nicotinate phosphoribosyltransferase
AEW60649.1	4	Dihydroorotase
AEW60161.1	4	methylthioribose kinase
AEW60328.1	3	dithiobiotin synthetase
AEW60284.1	3	quinolinate synthetase
AEW58915.1	3	endonuclease V
AEW62061.1	3	ProP effector
AEW59794.1	3	S-adenosylmethionine:tRNA ribosyltransferase-isomerase
AEW61679.1	3	riboflavin synthase subunit alpha
AEW60147.1	3	2,3-dihydro-2,3-dihydroxybenzoate dehydrogenase
AEW59335.1	3	4-hydroxyphenylacetate catabolism
AEW61865.1	3	integration host factor subunit alpha
AEW59764.1	3	hypothetical protein KPHS_10660
AEW60849.1	3	short-chain dehydrogenase/reductase SDR
AEW61939.1	3	peptidyl-tRNA hydrolase
AEW60154.1	3	putative alcohol dehydrogenase
AEW62352.1	3	GTP cyclohydrolase I
AEW63964.1	3	hypothetical protein KPHS_52660
AEW60098.1	3	putative aminotransferase
AEW60859.1	3	bifunctional indole-3-glycerol phosphate synthase/phosphoribosylanthranilate isomerase
AEW59902.1	3	inosine-guanosine kinase
AEW61823.1	3	cysteine desulfurase activator complex subunit SufB
AEW61142.1	3	hypothetical protein KPHS_24440
AEW63253.1	3	cystathionine beta-lyase
AEW61658.1	2	MarR family transcriptional regulator, transcriptional regulator for hemolysin
AEW60200.1	2	hypothetical protein KPHS_15020
AEW59806.1	2	transcription antitermination protein NusB
AEW58882.1	2	FMN reductase
AEW59807.1	2	thiamine monophosphate kinase
AEW63331.1	2	hypothetical protein KPHS_46330
AEW61876.1	2	transport protein TonB
AEW60629.1	2	transcriptional regulator, LysR family
AEW61859.1	2	putative outer membrane lipoprotein
AEW60868.1	2	short chain dehydrogenase
AEW62046.1	2	PTS enzyme IIC, mannose-specific
AEW61761.1	2	gluconate 2-dehydrogenase subunit gamma
AEW60470.1	2	arginine transporter permease subunit ArtQ
AEW59374.1	2	ferric iron reductase involved in ferric hydroximate transport
AEW62267.1	2	polysaccharide export protein
AEW60572.1	2	Methyltransferase

AEW63653.1	2	regulator of gluconate (gnt) operon
AEW64014.1	2	tRNA uridine 5-carboxymethylaminomethyl modification enzyme GidA
AEW60327.1	2	biotin biosynthesis protein BioC
AEW59479.1	2	thiamin/thiamine pyrophosphate ABC superfamily ATP binding cassette transporter, binding protein
AEW59895.1	2	DNA polymerase III subunits gamma and tau
AEW60654.1	2	hypothetical protein KPHS_19560
AEW58974.1	2	replicative DNA helicase
AEW60330.1	2	excinuclease ABC subunit B
AEW60387.1	2	hypothetical protein KPHS_16890
AEW62290.1	2	putative protease
AEW60848.1	2	catecholate siderophore receptor Fiu
AEW62503.1	2	Aminotransferase
AEW60555.1	2	23S rRNA m(2)G2445 methyltransferase
AEW59103.1	2	ribosome-associated GTPase
AEW63586.1	2	putative ATP-binding component of a transport system
AEW63683.1	2	RsmD family RNA methyltransferase
AEW60308.1	2	putative regulatory protein MerR
AEW60736.1	2	hypothetical protein KPHS_20380
AEW63329.1	2	putative enzyme, ferredoxin reductase-like, FAD-linked, NADP-linked
AEW62997.1	2	thymidylate synthase
AEW58722.1	2	coproporphyrinogen III oxidase
AEW63637.1	2	glycogen phosphorylase

* according to database GenBank accession: GCF_000240185.1

Supplementary Table 2. Exclusive proteins identified after 4 hours of native serum exposure. Proteins were identified using the software Patternlab, module AAPV, using a probability 0.01.

**Accession number ***	**Spectral count**	**Protein identification**
AEW63837.1	82	50S ribosomal protein L33
AEW63998.1	61	phosphate transporter subunit
AEW62851.1	35	iron ABC superfamily ATP binding cassette transporter, binding protein
AEW63565.1	17	50S ribosomal protein L4
AEW60387.1	17	hypothetical protein KPHS_16890
AEW63781.1	11	putative outer membrane protein
AEW61158.1	10	sensory histidine kinase DcuS
AEW63547.1	8	50S ribosomal protein L15
AEW62760.1	8	aldehyde dehydrogenase B (lactaldehyde dehydrogenase)

AEW60177.1	6	sugar ABC transporter, periplasmic sugar-binding protein
AEW61842.1	6	5-methyltetrahydropteroyltriglutamate-homocysteine methyltransferase
AEW59411.1	6	homoserine kinase
AEW58911.1	6	thiamin biosynthesis protein for pyrimidine moiety
AEW59722.1	6	phosphonate ABC superfamily ATP binding cassette transporter, binding protein
AEW61235.1	6	5-methyltetrahydropteroyltriglutamate--homocysteine methyltransferase
AEW63575.1	6	30S ribosomal subunit protein S12
AEW58782.1	5	50S ribosomal protein L31
AEW60492.1	5	translation initiation factor IF-1
AEW62691.1	5	phospho-2-dehydro-3-deoxyheptonate aldolase
AEW61869.1	4	hypothetical protein KPHS_31710
AEW59479.1	4	thiamin/thiamine pyrophosphate ABC superfamily ATP binding cassette transporter, binding protein
AEW59378.1	4	16S ribosomal RNA m2G1207 methyltransferase
AEW61628.1	4	alcohol dehydrogenase class III
AEW61679.1	4	riboflavin synthase subunit alpha
AEW62061.1	4	ProP effector
AEW62916.1	4	putative ABC-type transport protein
AEW60614.1	4	hypothetical protein KPHS_19160
AEW59410.1	4	bifunctional aspartokinase I/homoserine dehydrogenase I
AEW63467.1	3	30S ribosomal protein S9
AEW62466.1	3	lysine-arginine-ornithine-binding periplasmic protein
AEW59490.1	3	isopropylmalate isomerase small subunit
AEW60154.1	3	putative alcohol dehydrogenase
AEW59650.1	3	UbiE/COQ5 family methyltransferase
AEW59152.1	3	hypothetical protein KPHS_04540
AEW59058.1	3	putative DSBA oxidoreductase
AEW60544.1	3	nicotinate phosphoribosyltransferase
AEW63399.1	3	hypothetical protein KPHS_47010
AEW59027.1	3	ribose ABC superfamily ATP binding cassette transporter, binding protein
AEW60145.1	3	enterobactin synthase subunit E
AEW63089.1	2	flavodoxin
AEW62685.1	2	23S rRNA pseudouridine synthase D
AEW63545.1	2	30S ribosomal protein S13
AEW58910.1	2	thiamine-phosphate pyrophosphorylase
AEW59642.1	2	DL-methionine transporter substrate-binding subunit
AEW58926.1	2	homoserine O-succinyltransferase
AEW62862.1	2	hemin ABC superfamily ATP binding cassette transporter, binding protein HmuT
AEW58995.1	2	periplasmic maltose-binding protein
AEW59659.1	2	acyl-CoA dehydrogenase
AEW63671.1	2	high-affinity branched-chain amino acid ABC transporter periplasmic substrate-binding protein
AEW58927.1	2	malate synthase

AEW59806.1	2	transcription antitermination protein NusB
AEW60649.1	2	dihydroorotase
AEW61250.1	2	putative oxidoreductase
AEW61325.1	2	6-phosphogluconate dehydrogenase
AEW60146.1	2	2,3-dihydro-2,3-dihydroxybenzoate synthetase, isochroismatase
AEW61220.1	2	beta-lactamase SHV-11
AEW58928.1	2	isocitrate lyase

* according to database GenBank accession: GCF_000240185.1
